# A Multi-center Study on Improvement in Life Quality of Pediatric Patients with Asthma via Continuous Care

**Published:** 2017-11

**Authors:** Ying CAI, Junhua CAO, Ruixue KAN, Yuping LIU, Li ZHAO, Ming HU, Xuemei ZHANG

**Affiliations:** 1. Nursing Dept., Xuzhou Children’s Hospital, Xuzhou 221006, China; 2. Nursing Dept., The Affiliated Hospital of Xuzhou Medical University, Xuzhou 221002, China; 3. Nursing Dept., Xuzhou Center Hospital, Xuzhou 221009, China; 4. Nursing Dept., The First People’s Hospital of Xuzhou, Xuzhou 221002, China; 5. Nursing Dept., Xuzhou Maternity and Child Health Care Hospital, Xuzhou 221010, China

**Keywords:** Pediatric patients, Asthma, Continuous care, Multi-center

## Abstract

**Background:**

To analyze and summarize the effect of continuous care on the life quality and control of asthma of pediatric patients with asthma discharged from multiple hospitals.

**Methods:**

Retrospective analysis was carried out on 172 pediatric patients with asthma aged between 6 and 11 yr old randomly selected from those admitted to five hospitals between January 2014 and December 2015. Among these 172 patients, only 86 (intervention group) received the continuous care between January 2015 and December 2015, while the rest (control group) did not receive from January 2014 and December 2014.

**Results:**

After the patients in the intervention group were discharged from the hospital, the ratio of practical forced expiratory volume in one second (FEV1) to the expected FEV1 at the 12^th^ month was (90.28±10.35)%, and the ratio of peak expiratory flow to the expected value was (84.24±3.43)%, respectively higher than those [(82.73±8.86)% and (75.80±4.67)%] in the control group. Regarding pediatric asthma quality of life questionnaire (PAQLQ) between the intervention group and the control group, the difference had statistical significance (*Z*=−7.254, *P<*0.05). Childhood asthma control test (C-ACT) comparison between the intervention group and the control group indicated that the difference had statistical significance (*Z=*−7.918, *P<*0.05).

**Conclusion:**

Continuous care can improve the pediatric patient’s pulmonary function and life quality, and effectively control the asthmatic symptoms.

## Introduction

Continuous care, namely the nursing pattern that extends the in-hospital care to the discharged period through effective methods, includes a variety of methods, such as all kinds of network platform or telephone follow-up, which renders pediatric patients the access to the relevant nursing care in community or family (1–3). Children are a special population, pediatric chronic diseases of their own characteristics affected by various factors, like long disease course, susceptibility to recurrence and drug control. Out-hospital continuous care can prevent the recurrence of bronchial asthma (4).

The purpose of this study was to analyze and summarize the effect of continuous care on the life quality and control of asthma of pediatric patients with asthma discharged from multiple hospitals.

## Materials and Methods

This study was approved by the Ethics Committee of the hospitals. Signed written informed consents were obtained from the patients and/or guardians.

Inclusion criteria: 1) patients with definite diagnosis that conformed to the diagnostic criteria in *Guideline of Diagnosis, Treatment and Prophylaxis of Pediatric Bronchial Asthma* (5); 2) patients whose parents consented to participate in this study; 3) patients whose parents received the high-school education or above, and were able to negotiate. Exclusion criteria: 1) patients with generalized organic lesions; 2) patients complicated with other kinds of lesions in respiratory system, like allergic rhinitis or dysplasia in bronchi or lung; 3) patients with dysgnosia; 4) patients with other kinds of congenital diseases.

According to the inclusion criteria, we reviewed the files of pediatric patients between January 2014 and December 2014, and finally enrolled 86 patients meeting the requirement as the control group, in which there were 42 males and 44 females with an average age of (7.67±2.45) yr old; in January 2015, professional nurses of asthma mobilized the parents of pediatric patients that conformed to the inclusion criteria, wishing to invite their children to participate in the study; a total of 86 pediatric patients were finally involved in this study as the intervention group, in which there were 46 males and 40 females with an average age of (7.98±2.56) yr old. In comparison of gender and age, no statistically significant differences were observed between the intervention group and the control group (*P>*0.05), indicating that these data were comparable (Table 1).

**Table 1:** General data of pediatric patients with asthma in this study

***Age (yr)***	***Intervention group***		***Control group***		***Total***
	***Male***	***Female***	***Male***	***Female***	***%***
6–7	9	6	6	5	15.12
7–8	16	13	14	16	34.30
8–9	13	12	13	13	29.65
9–11	8	9	9	10	20.93
Total	46	40	42	44	100

### Research method

At the day of discharge, health file of pediatric asthma patient was established for each patient, in which the age, height, weight, contact information, address, history of disease and other relevant information of each patient were recorded. In addition, routine discharge instruction was also carried out, and all parents had access to the *Prophylaxis and Treatment of Pediatric Asthma*. Patients in the intervention group additionally received the continuous care based on the control group as follows:

### Method of continuous care

We organized a WeChat group of pediatric patients with asthma with the modern information technique. Profession nurses of asthma in each hospital were assigned to manage the WeChat group, and, in addition, attended the Health Management Training Courses of Diseases in Pediatric Respiratory System for 1 or 2 times in each year. Besides, in accordance with the relevant post instruction and assessment criteria, head nurses would carry out the assessment for these nurses per month to guarantee their homogeneity in nursing technique (6). In the WeChat group, physician-in-charge, clinical pharmacist in Respiratory Department, and experts of pediatric asthma were also invited. At discharge, patients’ parents could join the WeChat group through scanning the quick response code (QR code) under the guidance of professional nurses. Following services would be provided by the WeChat group: a) Information push: Health education on asthma would be delivered in text, picture, audio or video to the parents twice a week, and changes in climate would also be delivered to the parents in a real-time manner; additionally, information on expert outpatient service was also promptly released weekly to circumvent the parents to waste time in visiting the experts; content that was pushed in the WeChat group was composed by professional nurses, and released only after the approval of head nurses of Respiratory Department or Pediatric Department. b) Online inquiry: Between 15:00 and 17:00, professional nurses would review the questions and proposals of patients’ parents, and professional answers would be given promptly; for questions that were difficult to answer, nurses would record and deliver them to the nurses, clinical pharmacists, head nurses and experts for discussion, and the parents who proposed these questions would receive the answer later. c) Interactions: Patients and their parents were encouraged to share the experience and feelings in nursing care of asthma. d) Questionnaire: In the WeChat group, Childhood asthma control test (C-ACT) would be released, and parents were asked to answer the questionnaire in accordance with the requirement online; thereafter, nurses would be aware of the practical control of asthma of patients through reviewing the answers using a terminal equipment, and reminded these patients under poor control to seek for medical treatment in time.

### Telephone follow-up

For patients in the control group, telephone follow-up was performed only once within one week after discharge, aiming to comprehend the general condition of patients after discharge. For the patients in the intervention group, telephone follow-up was carried out once every ten days after discharge, and, one month later, the frequency was adjusted to once per month. The content of follow-up was recorded in the asthma file of patients, including the patient’s basic condition, awareness of the medication, efficacy, proper and improper living environment, answers to the question proposed by patient’s parents, informing the parents of the time of subsequent visit and the detailed information on appointment of subsequent visit. Following condition was also listed as the content of telephone follow-up: a) For patients who failed in subsequent visit, telephone follow-up should be carried out within 12 hours to figure out the reasons for failure in subsequent visit; b) Telephone follow-up should also be conducted for those patients whose parents raised the questions in the WeChat group that were not answered or resolved in time.

### Enriching the health lecture of asthma

Schemes on health lectures of asthma were stipulated, and these lectures were conducted in these 5 hospitals in turn. Patients’ parents would be informed of the time and address of the lectures held every month, and consulted to ascertain the participants and time. As for the lecture, we added the links of question and interaction, and the form was changed from the oral interpretation of nurses to the PowerPoint presentation prepared by professional nurses and experts who would introduce the induction factors, pathogenesis, pathogenic factors, treatment and nursing methods, and signs and treatment of asthmatic attack. After the lecture, lecturers would answer the question proposed by parents.

### Conducting the activity of summer camp in pediatric asthma patients

In the form of summer camp activity, we hoped to improve the relationship among pediatric patients, and that between the patients and medicals. A professional team called as *let children breathe freely* consisted of 5 physicians with the title of attending physician or above and 5 professional nurses of asthma from 5 hospitals, and invited the patients and their parents to attend the activities. Activities usually were held in the beautiful place that was capable of holding the physical training, and carried out in following forms: award question-answer of asthma. Patients and their parents who answered correctly obtained the delicate presents. To figure out the athletic ability of patients, we conducted the relay race, double dribble and treasure hunt. Furthermore, we examined the medications of each patient and evaluated the use of expiratory peak-velocimeter. Through those activities, we could precisely comprehend the activity status and inducing factors of each patient, and, accordingly, stipulated the exercise and rest schedules for patients. In this activity, 79 patients in the intervention group participated, and the rest patients failed for some reasons of family. Later, nurses would also stipulate the exercise and rest schedule according to the information of exercise provided by parents.

### Evaluation methods

Pulmonary function assay: At the 12 th month after discharge, we compared the ratio of practical forced expiratory volume in one second (FEV1%) to the expected FEV1 and that of peak expiratory flow to the expected value (PEF%), and the relevant data were obtained through review of the asthma file of patient.

Life quality of patient: Pediatric asthma quality of life questionnaire was performed respectively at the time of discharge and at 1, 3 and 12 months after discharge for evaluation of life quality through following aspects: symptoms (10 questions), feeling (8 questions) and exercise (5 questions). Scores were divided into 7 grades, and were in direct proportion to the life quality. Professional nurses were in charge of the questionnaire (7).

C-ACT: Assessment was carried out through C-ACT at 12 months after enrollment in both groups in form of questionnaire, in which 7 questions were put forward. Answer of each question was marked with the corresponding scores, and judgment on results was made according to the following criteria: total score not higher than 19 points for failure in control; total score between 20 and 22 points for partial control; total score not less than 23 points for control (8).

### Statistical methods

SPSS 24.0 (Chicago, IL, USA) was adopted in this study. Measurement data were presented as (*x̄*±s). Rank sum test was carried out in comparison of the control of asthma and life quality, and *a*=0.05 was set as the inspection level. *P<*0.05 suggested that the difference had statistical significance.

## Results

### Comparison of lung function between intervention group and control group

The ability of the forced expiratory volume for 1 second expressed as a percentage of the forced vital capacity (FEV1%) and peak expiratory flow (PEF) were significantly improved in intervention group than in control group at 12 months after discharge (*P*<0.05) (Table 2).

**Table 2:** Comparison of the pulmonary function between the two groups

***Group***	***Case (n)***	***FEV1%***	***PEF%***
Intervention group	86	90.28±10.35	84.24±3.43
Control group	86	82.73±8.86	75.80±4.67
t		3.78	5.37
P		<0.05	<0.05

### Comparison of the life quality indicators (Paediatric Asthma Quality of Life Questionnaire) between intervention group and control group

The quality of life was significantly improved in intervention group than in control group at 1 month, 3 months and 12 months after discharge (*P*<0.05) (Table 3).

**Table 3:** Scores of PAQLQ in the two groups

***Group***	***Case (n)***	***Excellent life quality n (%)***	***Basic life quality n (%)***	***Poor life quality n (%)***
Intervention group	86	59 (68.60)	20 (23.26)	7 (8.14)
Control group	86	16 (18.60)	25 (29.07)	45 (52.33)
Z		−7.254		
P		<0.05		

### Comparison of Childhood Asthma Control Test scores between intervention group and control group

Asthma Control Test scores were significantly improved in intervention group than in control group (*P*<0.05) (Table 4).

**Table 4:** Control of asthma in the two groups

***Group***	***Case (n)***	***Control n (%)***	***Partial control n (%)***	***Failure in control n (%)***
Intervention group	86	51 (59.30)	22 (25.58)	13 (15.12)
Control group	86	8 (9.30)	17 (19.77)	61 (70.93)
Z		−7.918		
P		<0.05		

## Discussion

Being put forward for the first time in 1947, continuous care has been gradually studied and defined by researchers ever since the 1980s. In 2001, Prossor et al. (9) proposed 6 dimensions in the concept model of continuous care, and, in 2003, Haggerty et al. (10) perfected the concept of continuous care on the basis of Freeman. In some countries, the pattern of continuous care has already been developed for a relatively long period, including the discharge-planning pattern (11) and nursing care pattern in transitional period. With the development in medicine, patients proposed more and more stringent requirement on nursing care (12). Ever since the continuous care has been applied in clinical practice, it has been providing more first-rate service for patients suffering from the chronic diseases, which has exhibited its increasingly important role (13). Individual management of pediatric patient with asthma could reduce the acute onset of asthma and the number of hospitalization (14).

Bronchial asthma is a kind of chronic disease frequently seen in childhood with the characteristics of chronic airway inflammation and bronchial hyperresponsivenes, and mainly characterized by the clinical features such as recurrent wheeze, cough, short of breath and chest distress. Additionally, complicated pathogenesis, susceptibility to recurrence and a variety of complications are also its features. For pediatric asthma patients, failure in long-term control and recurrent inhospital treatment will severely affect the prognosis of patients (15), e.g. the incidence of some diseases such as emphysema and pulmonary atelectasis has a significant casual relationship with the asthma. Thus, long-term self-management is required after discharge, in which there are differences on education level and awareness of the disease, inhalation drugs, use of expiratory peak-velocimeter and compliance. Literatures also have reported that ideal efficacy can hardly be attained in control through single medication, and that the long-term inhalation treatment at home and proper nursing intervention can remarkably improve the compliance of asthma patients to the medication (16–19).

In this study, we carried out a series of activities in forms of WeChat group, telephone follow-up, health lecture of asthma and summer camp of pediatric asthma patients for 86 pediatric asthma patients to help the patients and their parents form a proper understanding on asthma, resolve the puzzles of patients in familial nursing care, and render specific support, which could compensate the insufficiency in knowledge of drug administration, diet and exercise of patients and their parents, and improve the compliance and normality in medication. Through guiding and urging the patients to record the condition of asthma, we enhanced the self-management abilities of patients and their parents. Additionally, the confidence to conquer the disease was also strengthened via negotiation and communication between the patients, parents, and parents and medical staff, which is conducive to integrating the patients into group life and alleviating the negative feelings. Through comparisons, we found that at the 12th month after discharge, the FEV1% and PEF% in the intervention group were significant improved in comparison with the control group (*P<*0.05), and the PAQLQ scores and C-ACT scores in the intervention group were also remarkably higher than those in the control group (*P<*0.05), indicating that continuous care can improve the patient’s pulmonary functions and life quality, and effectively control the symptoms of asthma.

In this study, based on the individual management, five Three-A hospitals jointly carried out group management for pediatric asthma patients. During the management, how to improve the compliance of patient’ parents was the difficult point in this study. Thus, researchers, with the management form in other domains as model, adopted the merit points management in this study, in which they formulated the items and corresponding points. For example, in WeChat group, they put forward the *check-in* management system to remind the parents of the push information, and the points were given to the parents in following patterns: 1 point for 1 check-in; 3 points for participating in questionnaire; 5 points for sharing the nursing experience in WeChat group; 5 points for attending the health lecture; 5 points for attending the summer camp. Parents with 50 points or more would have free access to appointment with expert for once, and with 200 points or more would have free access to the examination of pulmonary function. Through the above measures, we aimed to promote the parents to participate actively in the activities, thereby guaranteeing the effectiveness of study.

## Conclusion

In addition to the achievement that has been gained in major medical organizations, especially the Three-A hospitals, continuous care can correct the imbalance in current development of medical system in China, which will render major pediatric patients, particularly those with chronic diseases, the access to the high-quality nursing care at home, and reduce the pains of patients and burdens on the family. Thu, continuous care has a promising future in development.

## Ethical considerations

Ethical issues (Including plagiarism, informed consent, misconduct, data fabrication and/or falsification, double publication and/or submission, redundancy, etc.) have been completely observed by the authors.

## References

[1] Paganelli F Spinicci E Giuli D (2008). ERMHAN: A Context-Aware Service Platform to Support Continuous Care Networks for Home-Based Assistance. Int J Telemed Appl, 2008: 867639. 10.1155/2008/867639 PMC2495075 18695739

[2] Qi GJ Chao YL Xi XY Liu KX Li WH (2015). Effect analysis of early bedside hemofiltration in treatment of severe pneumonia with acute renal failure of children. Eur Rev Med Pharmacol Sci, 19(24): 4795–4800. 26744871

[3] Rehm RS (2013). Nursing’s contribution to research about parenting children with complex chronic conditions: an integrative review, 2002 to 2012. Nurs Outlook, 61(5): 266–290. 23809600 10.1016/j.outlook.2013.03.008

[4] Au DH Macaulay DS Jarvis JL Desai US Birnbaum HG (2015). Impact of a telehealth and care management program for patients with chronic obstructive pulmonary disease. Ann Am Thorac Soc, 12(3): 323–331. 25642649 10.1513/AnnalsATS.201501-042OC

[5] Nishima S Furusho K (2003). New pediatric guideline for the treatment and management of bronchial asthma in Japan. Pediatr Int, 45(6):759–766. 14651560 10.1111/j.1442-200x.2003.01841.x

[6] Kvangarsnes M Torheim H Hole T Ohlund LS (2013). Intensive care unit nurses’ perceptions of patient participation in the acute phase of chronic obstructive pulmonary disease exacerbation: an interview study. J Adv Nurs, 69(2): 425–434. 22512673 10.1111/j.1365-2648.2012.06021.x PMC3594967

[7] Ehrs PO Larsson K (2004). Treatment improves quality of life in patients with poor perception of asthma. Prim Care Respir J, 13(1): 42–47. 16701636 10.1016/j.pcrj.2003.11.012 PMC6750654

[8] Liu AH Gilsenan AW Stanford RH Lincourt W Ziemiecki R Ortega H (2010). Status of asthma control in pediatric primary care: results from the pediatric Asthma Control Characteristics and Prevalence Survey Study (ACCESS). J Pediatr, 157(2): 276–281. 20472251 10.1016/j.jpeds.2010.02.017

[9] Prossor J (2007). Continuity of care. Br J Gen Pract, 57(545): 996. 10.3399/096016407782605036 PMC2084146 18252082

[10] Haggerty JL Reid RJ Freeman GK Starfield BH Adair CE McKendry R (2003). Continuity of care: a multidisciplinary review. BMJ, 327(7425): 1219–1221. 14630762 10.1136/bmj.327.7425.1219 PMC274066

[11] Hartigan EG Brown DJ (1985). Discharge planning for continuity of care. Program design: components and coordination. NLN Publ, (20-1977): 43–50. 3849709

[12] Clark A Nadash P (2004). The effectiveness of a nurse-led transitional care model for patients with congestive heart failure. Home Healthc Nurse, 22(3): 160–162. 15017316 10.1097/00004045-200403000-00005

[13] Price M Lau FY (2013). Provider connectedness and communication patterns: extending continuity of care in the context of the circle of care. BMC Health Serv Res, 13: 309. 23941179 10.1186/1472-6963-13-309 PMC3751828

[14] Bhaumik U Norris K Charron G Walker SP Sommer SJ Chan E Dickerson DU Nethersole S Woods ER (2013). A cost analysis for a community-based case management intervention program for pediatric asthma. J Asthma, 50(3): 310–317. 23311526 10.3109/02770903.2013.765447

[15] Xu JC Wu GH Zhou LL Yang XJ Liu JT (2017). Two unilateral puncturation comparative analyses of multiple-level fresh osteoporotic vertebral body compression fractures treated with percutaneous vertebroplasty guided by C-arm fluoroscopy or in senile patients. Eur Rev Med Pharmacol Sci, 21(7): 1456–1461. 28429363

[16] Huynh PN Scott LG Kwong KY (2010). Long-term maintenance of pediatric asthma: focus on budesonide/formoterol inhalation aerosol. Ther Clin Risk Manag, 6: 65–75. 20234786 10.2147/tcrm.s4025 PMC2835561

[17] Swerczek LM Banister C Bloomberg GR (2013). A telephone coaching intervention to improve asthma self-management behaviors. Pediatr Nurs, 39(3): 125–130, 145. 23926751

[18] Alados-Arboledas FJ Millan-Bueno P Exposito-Montes JF de la Cruz-Moreno J Perez-Parras A Arevalo-Garrido A (2011). [Safety and efficacy of continuous infusion propofol for diagnostic upper gastrointestinal endscopy in spontaneous breathing]. An Pediatr (Barc), 75(2): 124–128. 21429829 10.1016/j.anpedi.2010.11.012

[19] Toscani MR Makkar R Bottorff MB (2004). Quality improvement in the continuum of care: impact of atherothrombosis in managed care pharmacy. J Manag Care Pharm, 10(6 Suppl A): S2–12. 15546222

